# Comparison of a frequentist and Bayesian response-adaptive randomisation approach in multi-stage phase II selection trials with multiple experimental arms

**DOI:** 10.1186/1745-6215-16-S2-O58

**Published:** 2015-11-16

**Authors:** Christina Yap, Xuejing Lin, Ken Cheung

**Affiliations:** 1 Cancer Research UK Clinical Trials Unit, University of Birmingham, Birmingham, UK; 2 Mailman School of Public Health, Columbia University, New York, NY, USA

## 

The main objective of a phase II selection trial is to identify the most promising treatment amongst multiple competing experimental regimens when it truly exists, with moderate sample sizes.

Utilising response-adaptive randomization (AR) in such designs has ethical advantages as it steers patients away from the inferior treatment arms. A common approach uses Bayesian AR, whereby the randomization probability to an arm is based on the posterior probability that arm has the highest response rate. We also consider a frequentist alternative called sequential elimination (Levin 1981, Cheung 2008), which is a special form of AR. Using a simulation study based on an Acute Myeloid Leukaemia trial with four experimental arms, we compared the two approaches as well as a single stage pick-the-winner selection design (Simon et al, 1989). A Bayesian futility monitoring rule based on comparison to the historical response rate of standard treatment in such patients is also incorporated.

Under scenarios where all arms are futile, both AR designs perform better in terms of treatment selection and in-trial allocation than a single stage design. However, under scenarios where there is a winner, all approaches are comparable in terms of selection properties. Nevertheless, both AR approaches are more superior in allocating more patients to the best arm and are hence more ethical. The improved performance is more evident if there is a clear winner. The challenges of practical implementation of such approaches will be discussed, and one notable advantage of the Frequentist approach is that it is considerably more straightforward.

